# *fussel* (*fuss*) - A Negative Regulator of BMP Signaling in *Drosophila melanogaster*

**DOI:** 10.1371/journal.pone.0042349

**Published:** 2012-08-07

**Authors:** Susanne Fischer, Florian Bayersdorfer, Eva Harant, Renate Reng, Stephanie Arndt, Anja-Katrin Bosserhoff, Stephan Schneuwly

**Affiliations:** 1 Institute of Zoology, University of Regensburg, Regensburg, Germany; 2 Institute of Pathology, University of Regensburg, Regensburg, Germany; University of Dayton, United States of America

## Abstract

The TGF-β/BMP signaling cascades control a wide range of developmental and physiological functions in vertebrates and invertebrates. In *Drosophila melanogaster,* members of this pathway can be divided into a Bone Morphogenic Protein (BMP) and an Activin-ß (Act-ß) branch, where Decapentaplegic (Dpp), a member of the BMP family has been most intensively studied. They differ in ligands, receptors and transmitting proteins, but also share some components, such as the Co-Smad Medea (Med). The essential role of Med is to form a complex with one of the two activating Smads, mothers against decapentaplegic (Mad) or dSmad, and to translocate together to the nucleus where they can function as transcriptional regulators of downstream target genes. This signaling cascade underlies different mechanisms of negative regulation, which can be exerted by inhibitory Smads, such as daughters against decapentaplegic (dad), but also by the Ski-Sno family. In this work we identified and functionally analyzed a new member of the Ski/Sno-family, *fussel* (*fuss*), the Drosophila homolog of the *human functional suppressing element* 15 (*fussel*-15). *fuss* codes for two differentially spliced transcripts with a neuronal expression pattern. The proteins are characterized by a Ski-Sno and a SAND homology domain. Overexpression studies and genetic interaction experiments clearly reveal an interaction of *fuss* with members of the BMP pathway, leading to a strong repression of BMP-signaling. The protein interacts directly with Medea and seems to reprogram the Smad pathway through its influence upon the formation of functional Mad/Medea complexes. This leads amongst others to a repression of downstream target genes of the Dpp pathway, such as *optomotor blind* (*omb*). Taken together we could show that *fuss* exerts a pivotal role as an antagonist of BMP signaling in *Drosophila melanogaster*.

## Introduction

The TGF-β/BMP cascades control a wide range of developmental and physiological functions in vertebrates and invertebrates. The enormous array of cellular processes spans events from proliferation, differentiation, cell migration, angiogenesis to tumorigenesis, apoptosis and many more.

In *Drosophila melanogaster,* Decapentaplegic (Dpp), the fly homolog of BMP2/4, was one of the first ligands described within this signaling cascade [1], [2]. Already during eggshell patterning, Dpp has a direct long range function in order to specify the dorsal appendages and the operculum [3], [4]. In the early embryo it acts as a concentration-dependent morphogen involved in dorso-ventral patterning. In the wing, Dpp is forming a long-range gradient which determines longitudinal and crossvein position and orientation [5]. The wing itself and in particular its stereotypical array of veins has proven to be an attractive model system to unravel molecular mechanisms and interactions between proteins of many different signaling pathways, such as Hedegehog, Notch, EGFR, Wingless or BMP.

Due to the discovery of a growing number of ligands, the complexity of the *dpp* pathway has increased and is now referred to as the Bone Morphogenic Protein (BMP)/Activin-ß (Act-ß) cascade, as it can be divided in a BMP and an Act-ß branch [6]. Ligands of the BMP pathway involve Decapentaplegic (Dpp), Glass bottom boat (Gbb), a homolog of the vertebrate BMP5/6/7 [7], [8], and Screw (Scw), a more distantly related TGF-β protein [9]. All these ligands signal via the type I receptor Thick veins (Tkv) [10], [11] and/or Saxophone (Sax) [12], which both are recruited and phosphorylated through the constitutively active type II receptors Punt (Put) and wishful thinking (Wit) [13]. Likewise the Act-ß pathway is represented by several ligands, such as dActivin (dAct), Dawdle (Daw), Myoglianin (Myo) and Maverick. They have been described to interact with Put and Wit and the type I receptor Baboon (Babo) [14], [15].

The canonical BMP signaling from the cell-surface into the nucleus relies mainly on the Smad pathway (reviewed by [16]–[18]). Within this signaling pathway, Mothers against decapentaplegic (Mad), most homologous to the vertebrate Smad1 [19], functions as an activating R-Smad and is antagonized by the I-Smad Daughters against decapentaplegic (Dad), the homolog of Smad6 [20].

Within the Activin-ß cascade the activating R-Smad Smox, which reveals a high similarity to the vertebrate Smad3 and Smad2 has been described [21], [22]. For mediating downstream activation, R-Smads need to form a complex with the unique *Drosophila* Co-Smad4 Medea (Med), in order to exert their task as transcriptional regulator of corresponding target genes [23].

Like in other pathway networks, there is an enormous amount of extracellular and intracellular cross talk. It has been shown, for example, that the activation of Mad can be mediated by interplay of Dpp and Act-ß signaling, resulting in a trimeric complex of Mad/Smox/Medea as an alternative to the signaling through the dimeric complexes Mad/Med and Smox/Med [15].

Besides the above described members of the BMP/Activin-ß cascade, another family of genes, the *ski* and closely related *sno* (ski-related novel gene) genes are also involved in these signaling pathways. They were originally defined as oncogenes by their ability to transform chicken embryo fibroblasts upon overexpression and are widely described as potent negative regulators of the TGF-β cascade interacting for example with Smad2/3/4 in order to repress a multitude of target genes [24]. Yet their role in the mammalian system is complex. Pro- and/or anti- oncogenic activity of the Ski-Sno family depends on cancer tissues, stages of tumorigenesis, cell lines and the availability of complexes forming co-activators and co-repressors [25], [26]. In addition, the Ski complex seems to play an essential role in embryonic development repressing TGF-β-responsive promoters to a basal level [27], [28].

The *Drosophila* homolog of Sno, dSno, has been described independently by four different groups [4], [29], [30], [31]. Overexpression studies show, that dSno is involved in pathway switching from Dpp to Activin signaling and produces phenotypes reminiscent of loss of Dpp activity. The protein seems to be required during optic lobe development to maintain a proper balance between differentiation and cell proliferation [29]. Interestingly no homolog of the vertebrate Ski has yet been described in Drosophila.

In this study we report the discovery and functional analysis of a novel gene, the *Drosophila* ortholog of the human *functional suppressing element* 15 (*fussel-*15), to which we refer as *fussel* (*fuss*). The gene has been identified in an *in silico* screen as the Drosophila homolog of human *fussel-(15)* [32], a member of the Ski-Sno family in vertebrates. To study the function of Fuss in BMP/Act-ß signaling, we made use of GAL4 induced mis-expression in the Drosophila wing, an excellent model system to investigate BMP signaling. We could show that Fuss directly interacts with Medea, and hence interferes with the equilibrium state of R- and Co-Smads which leads, among other effects, to the inactivation of *dpp* target genes like *omb*. Therefore we assign *fuss* an important role as a negative regulator within the SMAD signaling cascade.

## Results

### Molecular and Phylogenetic Analysis of the Fussel Locus

**Figure 1:**
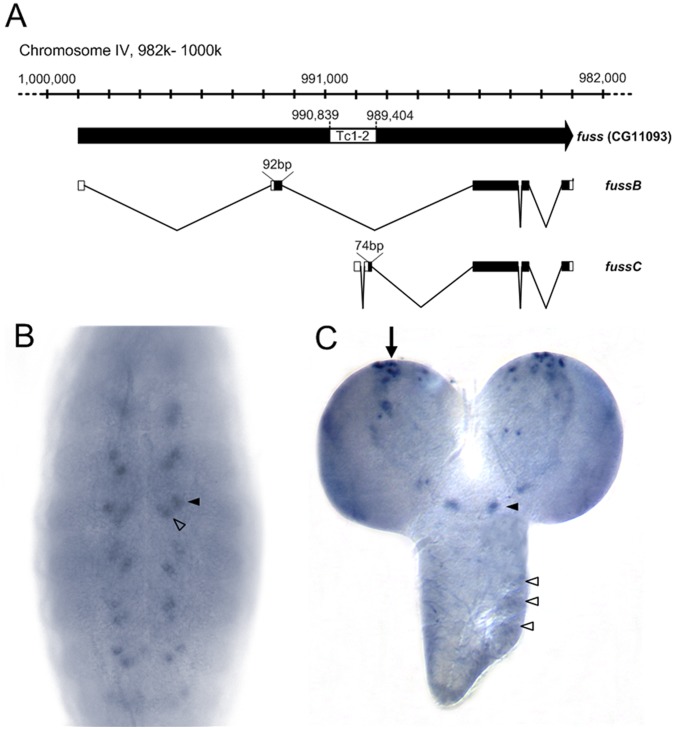
Genomic organisation of the *fussel* locus CG11093 and its transcription pattern. (A) 20 kb of the reverse complemented 102F4 cytological region of chromosome IV and the transcripts *fussB* (RefSeq NM_001169358.1) and *fussC* (RefSeq NM_001169359.1) are shown. The position of the Tc1-2- family transposon and the size of the two alternate exons are indicated. *In-situ* hybridisation of *fuss* shows expression in (B) two segmental clusters of cells in stage 14 embryos and (C) cells in the mediolateral (arrow) and SE- (arrowhead) and Tv-neuron-region (open arrowheads) in L3 brains.

*fussel* (*fuss*), annotated as CG11093, is localized at the cytological position 102F4 on the fourth chromosome, the smallest autosome of *Drosophila melanogaster* with about 1 Mb euchromatic region and nearly 80 genes [33]. Two annotated transcripts could be verified *in vivo* by means of RT-RCR, namely *fussB* (2571 nt) and *fussC* (2597 nt) (Fig. 1A). The insertion of a natural Tc1-2 transposon leads to a differential transcriptional start site and a variability at the N-terminus: the *fussC* transcript is coding for a protein with 84.2 kDa containing a unique 25 amino acid N-terminus. *fussB* encodes a 84.7 kDa polypeptide with an alternatively spliced N-terminus of 31 amino acids.

By means of *in situ* hybridization (ISH) experiments we could show that *fuss* is expressed in the embryo from stage 12 on (Fig. 1B). The expression pattern is restricted to a reiterated subset of 1–3 cells per hemineuromer in the CNS. In third instar larvae we find *fuss* expression in a neurogenic center within the medioposterior part of each brain hemisphere and also in cells in the suboesophageal ganglion (Fig. 1C). No substantial expression could be detected in wing or eye imaginal disks of third instar larvae. In agreement with the ISH experiments, quantitative RT-PCR revealed strong expression of *fussel* in the embryo, which decreases during larval stages and increases again within pre- and pupal stage (data not shown). These results are in full agreement with the data published by the modENCODE consortium [34] and suggest that Fuss is mainly required during embryonic and pupal development.

**Figure 2:**
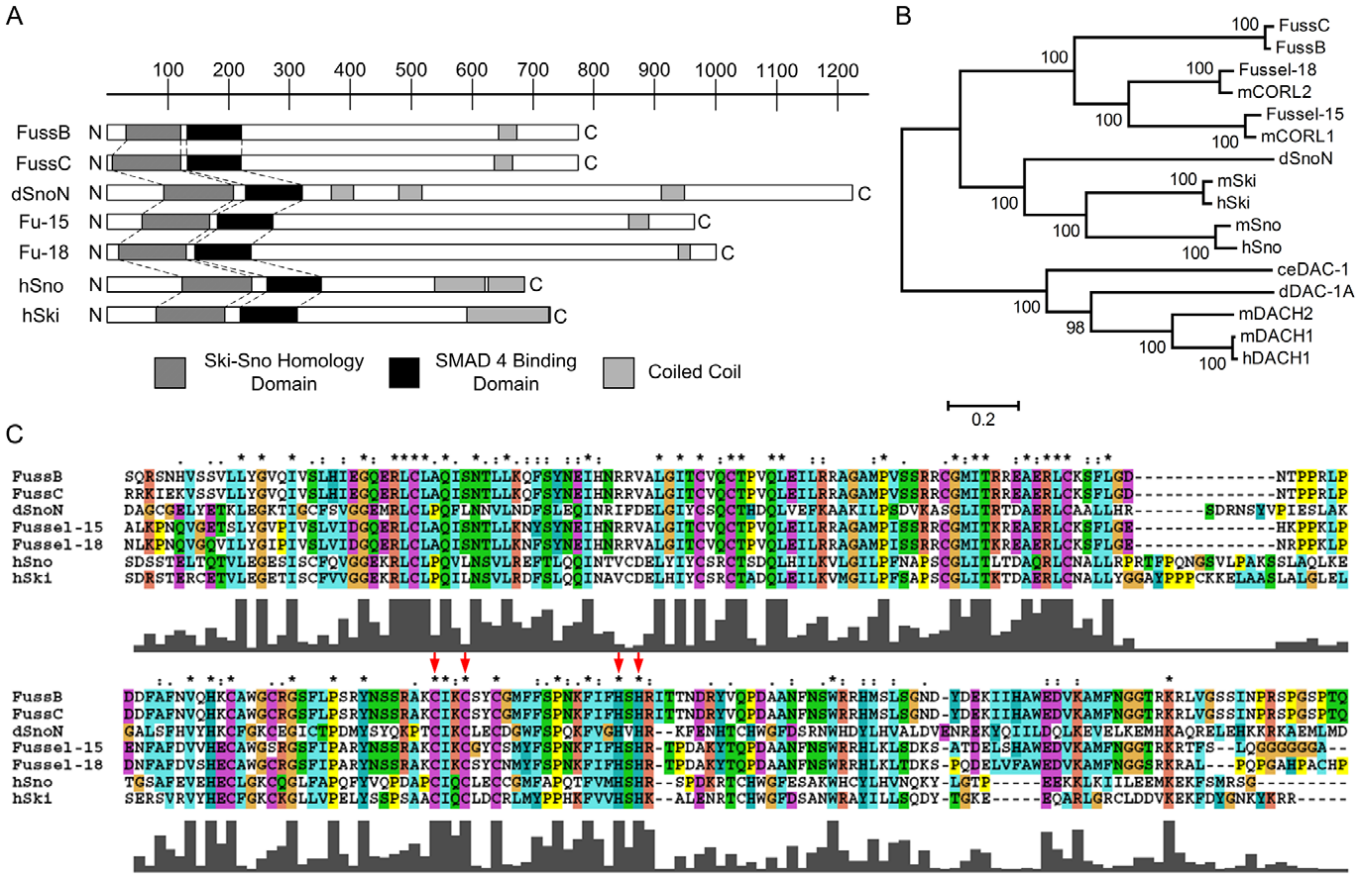
Fussel-Proteins and their relationship to the Ski-Sno/CORL/DACH-family. (A) Comparison of FussB and FussC proteins to Drosophila dSnoN-, human Fussel 15 and 18 and human Ski-Sno proteins. Shaded boxes show the relative positions of structural features as indicated in the legend; the scale-bar represents aminoacids. (B) Unrooted phylogenetic tree of the Ski-Sno/CORL/DACH- family. Branch length reflects phylogenetic divergence and the scale bar indicates the number of amino acid substitutions per site. Bootstrap values are given to indicate statistical significance at each node. (C) Multiple Sequence Alignment of the Ski-Sno-homology (upper alignment) and SMAD4- binding domains (lower alignment) of *Drosophila* and human Ski-Sno/CORL-proteins. Shading and the Clustal consensus indicates similar or identical amino acids. Height of bars reflects the quality of conservation while the symbols are denoting the conservation type: stars mark identical or conserved residues in all sequences while colons and dots indicate conserved or semi-conserved substitutions. Red arrows mark the zinc binding domain.

The Fuss protein itself is characterized by a Ski-Sno homology domain at the N-terminus, which is present in all members of this proto-oncogene group (Fig. 2A). The domain shares a characteristic feature with members of the Dachshund family which is known as Dac-box, DS or DHD. It is present in worms, flies and mammals, resulting in an integration of Fuss within the Dach-subfamily (Fig. 2B). The folding pattern of this domain contains a helix turn helix and a beta-alpha-beta turn motif, suggesting a putative DNA binding activity.

The second domain with significant amino acid identity is the SMAD4 binding domain, which shares structural homology with the SAND domain, named after Sp100, AIRE-1, NucP41/75, DEAF-1 (Fig. 2A). It represents an evolutionarily conserved sequence motif found in nuclear proteins, which are involved in chromatin-dependent transcriptional regulation [35]. Interestingly only the N-terminal part of this domain shows high homology among the Ski-Sno members. Within this domain four residues Cys173, Cys176, His188 and His190 coordinate a bound zinc atom, contributing to the structural stability (arrows in Fig. 2C). These residues are a characteristic feature for the zinc binding ability of the Ski-family [36]. Finally, a coiled-coil region with a leucin zipper-like motif can be identified at the C-terminus, indicating a functional oligomerization or protein-protein interaction of Fussel (Fig. 2A; [36]).

Phylogenetic analysis of the protein on the basis of the neighbour joining method elucidates the relationship between the two *Drosophila* and *human* Fussel proteins, *Drosophila* dSnoN, the human and mouse Ski-Sno members and the Dachshund family (Fig. 2B). The vertebrate Ski-Sno family has a significant homology to the human and *Drosophila fussel* proteins. Their biological function is diverse, ranging from involvement during embryonic development of muscle, central and peripheral nervous systems or respiratory tissue to regulation of growth and differentiation of adult tissues like neural, muscle or hematopoetic cells. This group of proteins is known for its pro-oncogenic function due to their ability to antagonize the growth-inhibitory activity of the TGF-β/Smad pathway [37], [27]. From the phylogenetic tree it becomes obvious, that FussB and FussC form a homophyletic group with h-FUSSEL-15 and 18, also known as Ladybird homeobox corepressor 1 (LBXCOR1) and Ladybird homeobox corepressor 1-like protein CORL2, and their homologues in mice, mCORL1 and mCORL2 [32], [38]–[41]. This group has also been described as the CG11093 or Iceskate family [32], [42] with *fu*ss as its unique member in *Drosophila*.

### *fussel* Acts as an Antagonist within the BMP Pathway

**Figure 3:**
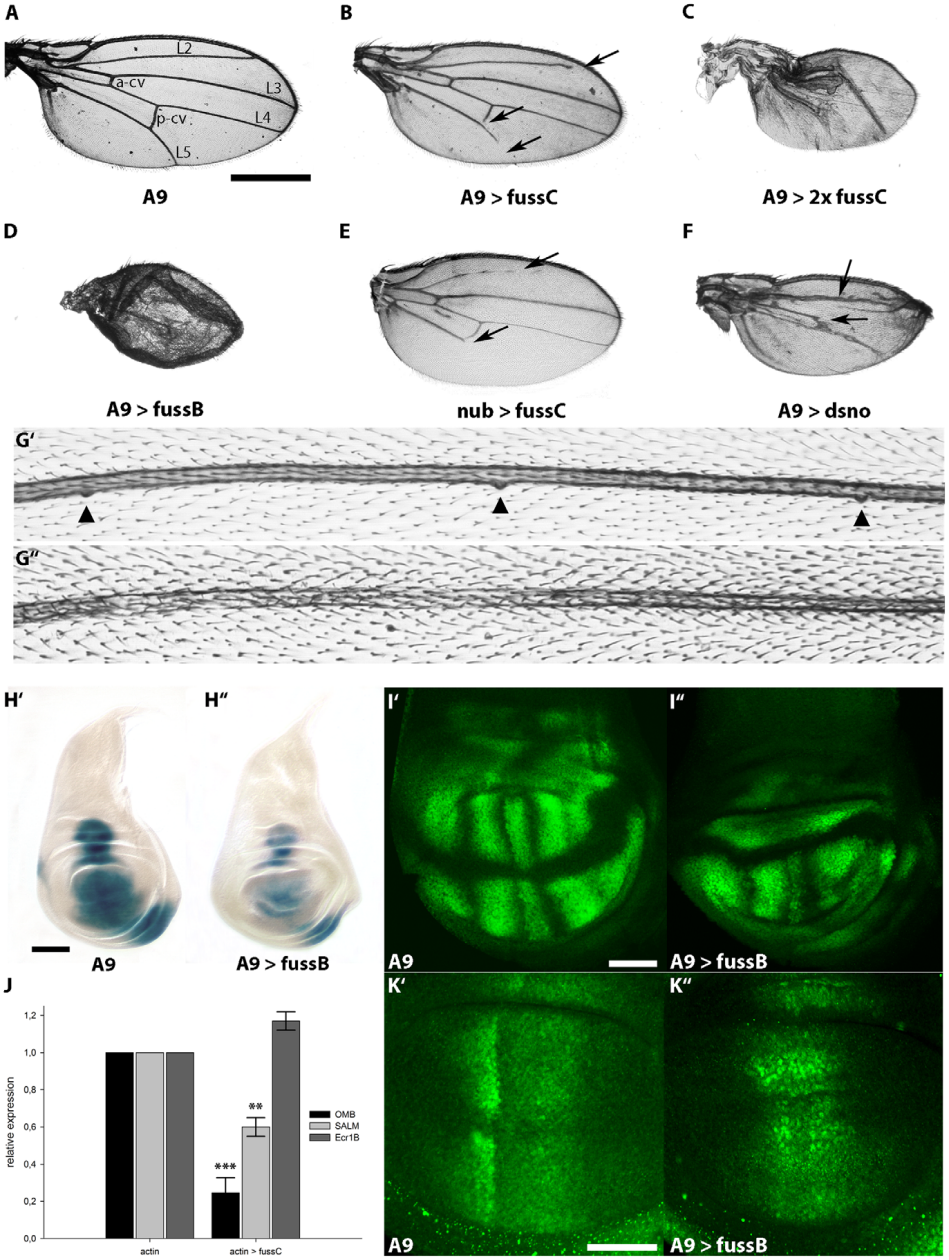
Ectopic expression of *fussel* in the wing disc reduces wing size, leads to loss of veins, loss of campaniform sensilla and interferes with the expression of BMP target genes. (A) Control wing of a male fly from the *A9-Gal* line. Longitudinal (L2 to L5) and cross veins (a-cv, p-cv) are indicated. (B) *A9-Gal4; UAS-fussC*. The wing is smaller, arrows indicate the truncation of L2, L5 and the p-cv. (C) *A9-Gal4; UAS-fussC/UAS-fussC*. Expression of two copies of *UAS-fussC* enhances the observed phenotype. (D) *A9-Gal4; UAS-fussB*. Expression of one copy of *fussB* leads to a reduction of wing size and a severe disruption of the overall wing structure. (E) *nub-Gal4; UAS-fussC*. The L2 and L5 veins are truncated. (F) *A9-Gal4; UAS-dSno*. Expression of *dSno* leads to a reduction of wing size and a loss of the L4 vein. (G) Mis-expression of *fuss* leads to loss of campaniform sensilla. (G’) Medial part of the L3 vein of a male *A9-Gal4* fly. Three campaniform sensilla are marked with arrowheads (G”) *A9-Gal4; UAS-fussC*. Distal to the p-cv, all campaniform sensilla are lost. (H) *fuss* represses *omb* expression. Micrographs of X- Gal- stained female L3- wing discs: (H’) *omb-lacZ; UAS-fussB*; (H”) *omb-lacZ/A9-Gal4*; *UAS-fussB/+.* (I) The *blistered* (dSRF) domain in male L3-wing discs is disrupted by *fuss* expression. Confocal scans of (I’) *A9-Gal4*; (I”) *A9-Gal4; UAS-fussB.* (J) Relative expression of *omb*, *salm* and *ecr1b* in *actin-Gal4/fussC* L3-larvae compared to *actin-Gal4* controls. Asterisks indicate the level of statistical significance (t-test **p<0.01, ***p<0.001). (K) *fuss* disrupts the pattern of activated Mad but does not inhibit its phosphorylation. Confocal scans of anti-phospho-SMAD1/5 stained L3-wing discs: (K’) *A9-Gal4*; (K”) *A9-Gal4; UAS-fussB*. Scale bars represent 500 µm (A), 100 µm(H’) and 50 µm(I’, K’).

As already introduced, the *dpp* pathway represents a homolog to the vertebrate BMP2/4 cascade and its signaling is accomplished by proteins, which are widely conserved. One member of the Ski-Sno family in Drosophila is *dSno*, described to exert an inhibitory function within the BMP and Activin-ß signal transduction pathways [29]–[31]. Hence *fuss,* due to its structural similarity (see Fig. 2A), might also act as a transcriptional regulator within the *dpp* pathway. Due to the lack of *fuss* mutants (fourth chromosome localization) we used a reverse genetic approach to characterize the function of *fuss*. The expression of available *fuss* RNAi constructs from the National Institute of Genetics in Japan and from the Vienna Drosphila RNAi Center with *actin-Gal4* (*act-Gal4*) or *daughterless-Gal4 (da-Gal4)* revealed conflicting results with either no phenotype at all or pupal lethality and possible off-target effects. In order to initiate the analysis of the function of Fussel on this pathway, two overexpression lines were established containing either *fussC* or *fussB* full length cDNAs. We first overexpressed *fuss* via *A9-Gal4* in the wing, a favored system to study novel components of the *dpp* cascade, as morphological changes in wing shape, size and vein formation of the adult fly can easily be discovered. The overexpression of one *UAS-fussC* copy with *A9-Gal4* leads to vein truncations of L2, L5 and p-cv at 100% penetrance (Fig. 3B) and results in a smaller wing size (75% of the control flies; compare Fig. 3A to 3B). Expressing two copies of *fussC* causes wing size reduction up to 46% and a dramatic loss of vein and intervein tissue (Fig. 3C). Stronger phenotypes become manifested in unfolded wings (data not shown). Likewise the driver of *nubbin (nub-Gal4)*, a gene expressed in the wing pouch cells of the wing disk [43], provokes defects in L2 and L5 which, similar to the overexpression with *A9-Gal4,* do not reach the distal margin of the adult wing (Fig. 3E). An even more conspicuous phenotype can be monitored, when the *fussB* form is driven with *A9-Gal4*. The male flies are lethal in the late pupal stage and the very few female escapers show a strong disruption in wing development. The wings are smaller (27% compared to the driver line) lacking veins and are poorly unfolded (Fig. 3D). This can be ascribed to the fact, that the *UAS-fussB* construct contains an optimized Kozak consensus sequence, resulting in stronger overexpression phenotypes than the ones observed with *UAS-fussC*. Nevertheless, we could show that both proteins do not differ in their physiological function, as a construct carrying the optimized Kozak sequence 5′ prime to the translation start of *fuss-C*, instead of its endogenous one, phenocopies *UAS-fussB* overexpression in the wing (data not shown). For our experiments this difference is of great advantage, as the *UAS- fussB* results in lethality when expressed in the wing (see above), hence we used the *UAS-fussC* form to investigate interactions in the adult wing and *UAS-fussB* to monitor more clearly phenotypes in the larval disks (see below).

Although ectopic expression of *dSno* also negatively interferes with BMP/Activin-ß signaling [29], the wing phenotype is distinct. In the wings of *A9-Gal4;UAS-dSno* flies L4 is lost from the p-cv on towards the wing margin and L3 can be identified, but only rudimentary and in a stunted growth (Fig. 3F). This indicates, that the two genes differ in the way they exert their effect as antagonists of the *dpp* cascade. Furthermore we observe in *A9-Gal4;UAS-fussC* wings a loss of mechanosensory neurons, the campaniform sensilla located on L3 (Fig. 3G’/G”).

The modulation of vein development described above focused our interest on possible downstream regulating effects of *fuss* upon the BMP/Activin-ß cascade. The most prominent readouts of *dpp* signaling are the expression of *optomotor blind* (*omb*) and *spalt major* (*salm*) along the anterior/posterior (A/P) boundary of the imaginal wing disk [44], [45]. Imaginal wing disks of third instar female *omb-lacZ*; *UAS-fussB* larvae show a wild type *omb* expression pattern along the anterior-posterior compartment boundary and in the mediolateral regions of the disk (Fig. 3H’). Yet, when *fussB* is mis-expressed using the *A9-Gal4* driver in the *omb-lacZ* background, we can monitor a clear reduction of *omb* expression within the entire disk of female larvae (n = 15). The center and part of the dorsal wing pouch compartment where *A9-Gal4* is expressed shows the strongest decrease of the staining, strengthening the role of *fuss* as a negative regulator. In addition, the dorsal and ventral wing hinge regions are also affected (Fig. 3H”).

Quantitative RT-PCR with third instar larvae confirms our histological observations. The level of *omb* mRNA is significantly reduced to 25% (Fig. 3J). Furthermore, *salm,* whose expression in the wing blade is strictly dependent on *dpp* signaling and which covers a broad central domain from the L2 provein to the anterior limit of the L5 [46], is reduced to 60% (Fig. 3J). This shows that *fuss* is able to negatively interact with BMP signaling *in vivo* and is consistent with its proposed role as an inhibitor. We also tested a known readout of the Activin pathway, namely the expression of the Ecdyson Receptor 1b (*EcR1b*; [47]). In contrast to the clear downregulation of *dpp* signaling transcription targets *omb* and *salm*, we did not observe any significant changes of *EcR1b* relative expression (Fig. 3J).

To further assay the modulating effects of *fussel* on BMP signaling and vein patterning we examined the expression of the *Drosophila* Serum Response Factor (DSRF) in third instar larval imaginal disks. The expression of DSRF protein is restricted to the wing pouch and the hinge region, representing the future intervein tissue and is repressed by *dpp* signaling [48]. Controls show that the DSRF protein is absent from the future wing margin and the area of the prospective wing veins (Fig. 3I’). *A9-Gal4;UAS-fussB* larval disks reveal irregular DSRF expression with almost complete absence of the protein in the notably reduced dorsal part and a highly irregular pattern in the ventral part lacking clear vein primordia regions (Fig. 3I”).

As intracellular signaling of BMP in *Drosophila* is accomplished through the vertebrate R-Smad homolog Mad, we investigated if Fuss affects the phosphorylation or localization of Mad in the third instar wing disk. PMad concentration is a direct measure of the Dpp signaling activity [49], [50] and can be assayed by use of an anti- phospho-SMAD1/5 antibody, which detects endogenous pMad in two prominent stripes (Fig. 3K’) [51], [52]. When overexpressing *fussB* with *A9-Gal4* the pMad staining persists, but clearly deviates from the sharp borders of activated Mad observed in control imaginal disks (Fig. 3K”). This indicates that Fuss does not repress Mad activation, but rather leads to a reallocation of Mad within the wing disk. Moreover, having a closer look at the morphology of *A9-Gal4;UAS-fussB* larval wing disks, a notable reduction of the dorsal compartment becomes obvious (Fig. 3H”, I”, K”). In consequence, despite the almost normal larval wing disk size (Fig. 3H”), the abnormal adult wing observed in fig. 3C and D can now be explained, by taking into account that the vein-to intervein ratio is also affected during metamorphosis.

### Genetic Interaction of *fussel* with Members of the BMP Pathway

**Figure 4:**
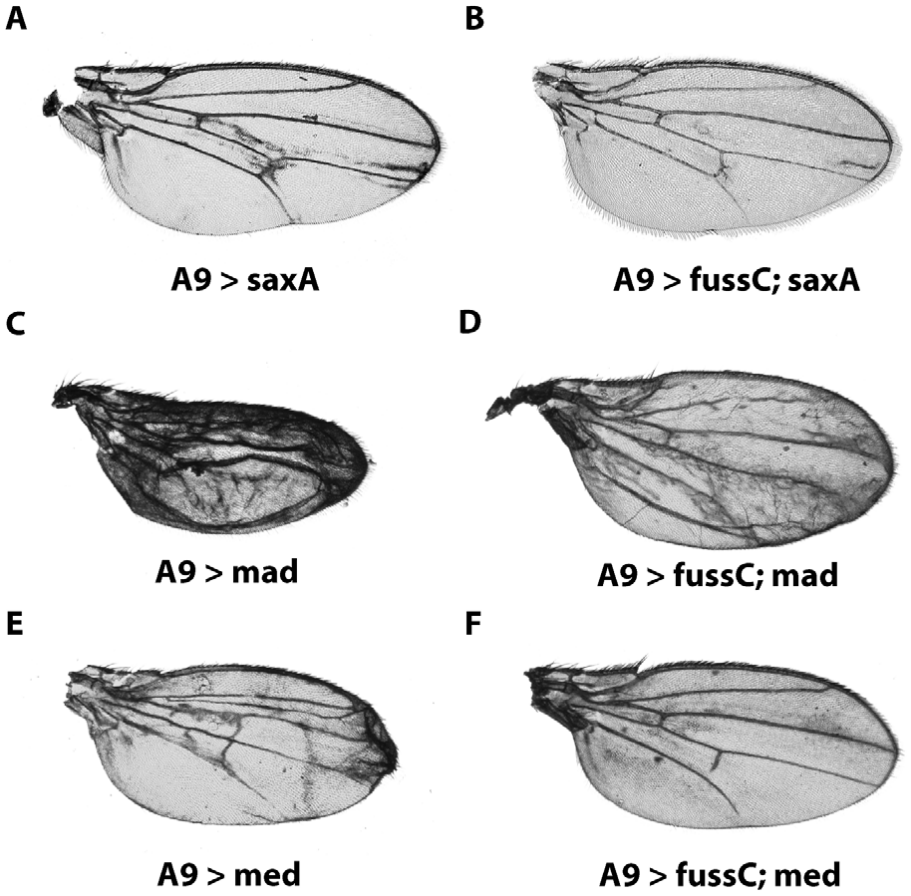
Genetic interaction of fussel with members of the BMP pathway. (A) *A9-Gal4/y; UAS-saxA*. Overexpression of constitutively active *sax* causes growth of additional vein material between L3–L5 (B) *A9-Gal4/y; UAS-saxA; UAS-fussC*. Coexpression of *fussC* ameliorates vein overgrowth. (C) *A9-Gal4/y; UAS-mad*. Overexpression of *mad* transforms most of the intervein tissue into vein tissue and eventually results in a blistered wing. (D) *A9-Gal4/y; UAS-mad; UAS-fussC*. Coexpression of *fussC* ameliorates blistering and considerably improves vein pattering. (E) *A9-Gal4/y; UAS-med*. Overexpression of med causes distinct overgrowth and dublication of wing veins. (F) *A9-Gal4/y; UAS-med; UAS-fussC*. Coexpression of *fussC* rescues the vein overgrowth and restores the *fussC*-phenotype.

Due to the specific effects of *fuss* on *dpp* dependent target genes we performed genetic interaction experiments in the wing with wild type, constitutively active or dominant negative forms of various members of the BMP/Activin-ß pathways. Saxophone, a type I serine-threonine receptor of the pathway [12] mediates signaling from Dpp and Gbb for patterning the wing imaginal disk (reviewed by O’Connor *et al.* 2006 [5]). Constitutively active Saxophone (SaxA) functions independently from endogenous signals [53] and its overexpression with *A9-Gal4* leads to extra vein material between the region of L3 and L5 with slight wing blistering, also affecting the p-cv pattern (Fig. 4A). Interestingly, introducing one copy of *UAS-fussC* in these flies, the phenotype is almost completely rescued and the vein formation is close to wild type (Fig. 4B), suggesting a suppressive effect of *fuss* on the BMP pathway. In the case of Mad, an overexpression with *A9-Gal4* leads to a dramatic reduction in wing size, ectopic vein tissue and blistering (Fig. 4C). Coexpression of Fuss restores not only the size of the wing, but leads also to a notable amelioration of the vein-, respectively intervein patterning (Fig. 4D). Similar to Mad, Fuss is able to restore wing shape and vein patterning of Med overexpression to an almost wild type mode, with only slight failure of complete outgrowth of L5 and p-cv, reminescent of the *fussC* phenotype (Fig. 4E, F). These data reveal that in the wing, *fuss* is able to interfere negatively with the BMP activity gradient. Mad is known to form a complex together with Med in order to actively translocate to the nucleus and transduce *dpp* signaling [54]. To this point we can not conclude if the interaction is based on a complex formation of Fuss with either Med or Mad, or if the effects we observe in the wing are due to titration effects, where Fuss for example sequesters Med, inhibiting the Mad/Med complex to perform its transcriptional functions.

For the analysis of interactions with the Activin-ß cascade, we also investigated the coexpression of *fuss* with *baboon*, the type I receptor [14] and its associated mediator *dSmad2*. Neither the three different *baboon* isoforms nor two copies of *dSmad2* show any vein defects by themselves when overexpressed with *A9-Gal4* [15]. Therefore it is not surprising that additional coexpression of *fussC* leads merely to a modest *fuss* phenotype, resulting in a slight truncation of L5 and a minor reduction in wing size but no signs of interaction (data not shown). Expression of a constitutively active form of *baboon* (UAS-babo*) leads to tissue overgrowth in the wing and patterning defects in the veins [22]. However, in contrast to *SaxA* we could not observe any modification of this phenotype after co-overexpressing *fuss* in the wing (data not shown), which leads us to the conclusion, that *fuss* does not directly interact with the Activin-ß pathway. This confirms our data from the quantitative RT-PCR experiments, where we could not monitor any changes of *EcR1b* expression (Fig. 3J), a readout of the Activin-ß pathway.

Taken together, our results indicate that *fuss* exhibits its function as a negative regulator of the BMP cascade, most likely through interaction with the activating Co-Smad Medea.

### Nuclear Translocation of Fussel through Medea

The presence of a SMAD binding domain in the Fuss Protein and the results we obtained from the genetic interactions provoked us to further study the interaction between Fuss and Med, respectively Mad, on a molecular level by a direct yeast two hybrid experiment. The *fuss* cDNA was cloned *in frame* with the GAL4DB of the bait vector pDBLeu, *mad* and *med* were inserted downstream of the pPC86 GAL4 activation domain.

**Figure 5:**
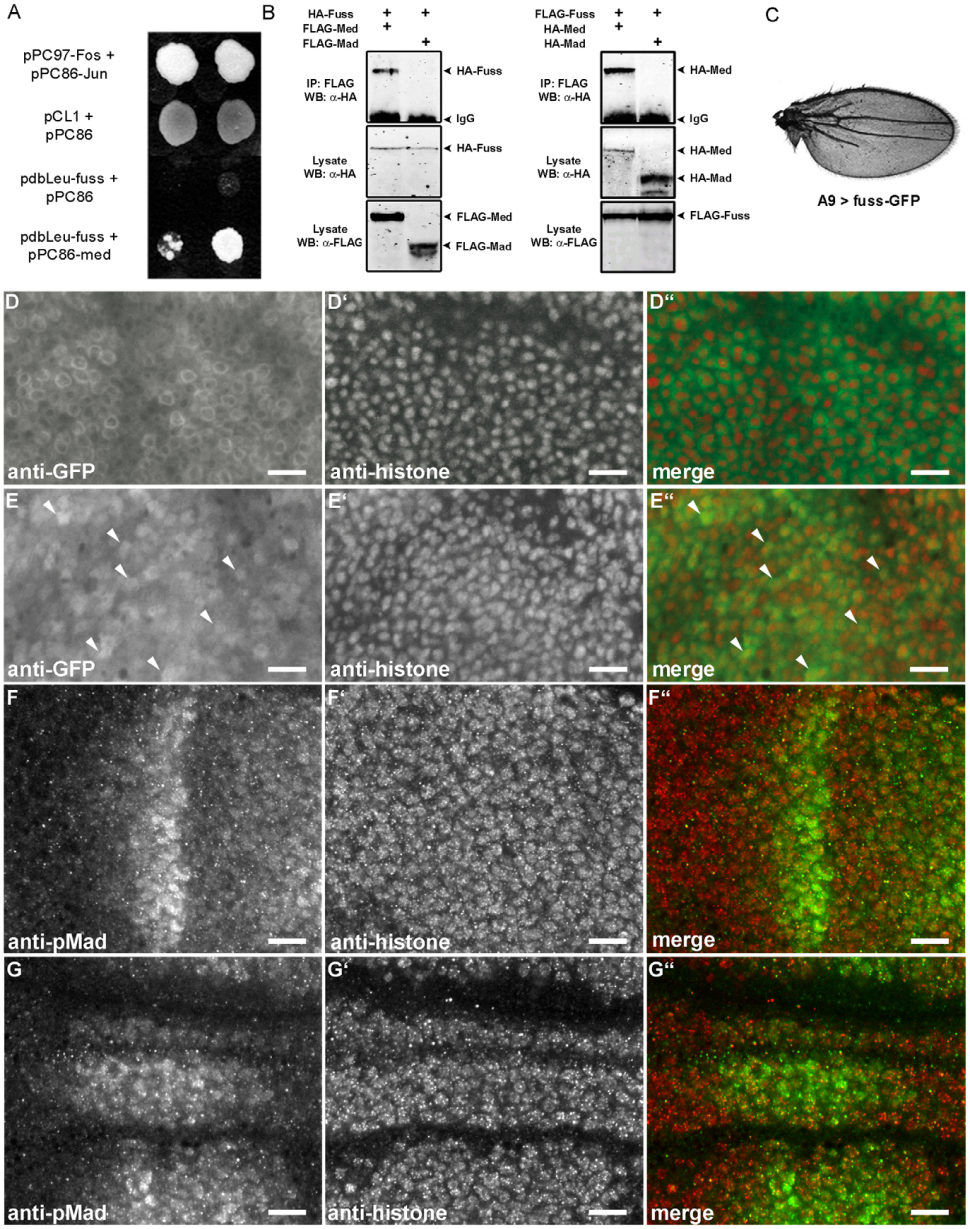
Formation of a Fuss/Med protein complex and its translocation into the nucleus. (A) Yeast Two Hybrid experiment showing physical interaction of Fuss and Med: *pPC97-Fos + pPC86-Jun*: interaction control; *pCL1+ pPC86*: Gal4- growth control and empty prey vector; *pdbLeu-Fuss + pPC86*: negative control; *pdbLeu-Fuss + pPC86-Med*: positive interaction of Fuss and Med. (B) Coimmunoprecipitations: in lysates from S2-cells co-expressing HA-Fuss together with FLAG-Med or FLAG-Mad, HA-Fuss co-precipitates with FLAG-Med but not with FLAG-Mad. In lysates from S2-cells co-expressing FLAG-Fuss with HA-Med or HA-Mad, HA-Med co-precipitates with FLAG-Fuss and HA-Mad does not. (C) Wing of a male fly of the genotype *A9-Gal4; UAS-fuss-GFP*. Overexpression of *fuss-GFP* leads to truncations of L2 and L5 veins and a loss of the p-cv. (D–E”) Upon co-expression of Med, Fuss-GFP is partially translocated into the nucleus while the cytoplasmic fraction of the protein is reduced. Confocal scans of L3 wing discs stained with anti-GFP (D, E) and anti-Histone (D’, E’) antibodies. (D–D”) *A9-Gal4; UAS-fuss-GFP*. The Fuss-GFP fusion protein is mainly localised in the cytoplasm. (E) *A9-Gal4; UAS-fuss-GFP; UAS-Med*. Arrows emphasize some of the cells that clearly show nuclear localisation of the Fuss-GFP fusion protein. (F–G”) Fuss does not inhibit the nuclear translocation of pMad. Confocal scans of L3 wing discs stained with anti-phospho-SMAD1/5 (F, G) and anti-Histone (F’, G’) antibodies. (F–F”) *A9-Gal4.* (G–G”) *A9-Gal4; UAS-fussB*. All Scale bars: 10 µm.

The direct interaction of *fussel* with *med* could be clearly monitored through growth tests on the accordant selective media in four independent sets of experiments (Fig. 5A) and does not result from self activation of *fuss* with the bait vector (5A: pdbLeu-FussC+pPC86). However, we could not verify an interaction of Fuss with Mad on an *in vitro* level (data not shown). In addition we performed co-immunoprecipitations (CoIPs), for which HA and FLAG tagged *fuss-*, *mad-* and *med-* cDNAs were co-expressed in *Drosophila* S2 cells. Analysis of the cell lysates showed, that Fuss and Med do bind to each other whereas Fuss and Mad do not. To rule out effects that could result from the protein fusions with the HA/FLAG epitopes, we repeated the experiment with exchanged tags and obtained the same result (Fig. 5B). Taken together, these observations indicate that Fuss specifically interacts with Med to exert its inhibitory functions.

However, what is the cellular consequence of a Fuss/Med interaction and what are the possible reasons for a downregulation of Dpp target genes? On the one hand there is the possibility of Fuss/Med heterodimer degradation in the cytoplasm, on the other hand, the heterodimer could translocate to the nucleus and act as a transcription-complex altering gene expression. To gain insight into the molecular processes initiated through the binding of Fuss to Med, we decided to study the subcellular localization of Fuss. First we established a *fuss*-eGFP line. The 105 kDa protein carries the GFP tag at the C-Terminus and is fully functional: overexpression with *actin-Gal4* is lethal in pupae and the wing phenotype with *A9-Gal4* can even be considered as an enhancement to the one we observe with the *fuss*C cDNA, namely a stronger reduction in wing size to 62% of wild type and a more severe vein loss of L2, L5 and p-cv (Fig. 5C). This might be a result of the GFP fusion which could lead to an increase of the Fuss protein stability. Confocal analysis of GFP staining in third instar wing discs shows an almost exclusive localization of the protein in the cytoplasm, becoming most apparent dorsal to the wing blade margin (Fig. 5D–D”). However, when *fuss*-GFP and Med are co-expressed with *A9-Gal4,* we detect a noticeable displacement of the GFP signal to the nucleus (Fig. 5E–E”). As already mentioned, the R-Smad Mad usually forms a complex with Med in order to fulfill its role as a transcriptional regulator in the nucleus [23]. Knowing that Med influences the localization of Fuss, we wanted to study possible effects of Fuss on Mad localization, too. We analyzed the subcellular localization of pMad in the dorsal compartment of L3 wing disks. In control disks pMad accumulates in a sharp stripe of cell nuclei (Fig. 5F–F”). Although the overall distribution of pMad in *A9-Gal4; UAS-fussB* disks is changed, there is no noticeable difference of the subcellular pMad localization (Fig. 5G–G”). Alltogether, these results indicate that Fuss is able to bind to Med in the cytosol and to translocate into the nucleus, either as a Fuss/Med complex, or as part of a Mad/Medea complex.

## Discussion

In this report we have characterized a new gene in *Drosophila melanogaster*, *fussel* (*fuss*), an ortholog of the human functional smad suppressing element 15 (*fussel*-15), also known as SKOR1, Corl1 or LBXcor1 (UniProtKB ID: P84550). Fuss is characterized by a Ski-Sno and a SAND homology domain and can be classified as a proto-oncogene. The two transcripts, *fussB* and *fussC*, diverge in the N-terminus and represent two phylogenetically different versions of the gene: *fussB* is the original form of the CG11093 locus, whereas the *fussC* transcript is spliced differently due to the subsequent integration of a Tc1-2 transposon (Fig. 1A). The ubiquitous mis-expression of both forms is lethal in pupal stages. Its endogenous expression pattern during embryogenesis and larval development is neuronal, which is similar to the vertebrate genes *fussel-15* and *fussel-18* which also show a restricted pattern of expression mainly limited to neuronal tissue such as the developing murine cerebellum and the spinal cord [32], [38], [41].

As Ski-Sno proteins are described to repress TGF-β signaling through their interaction with Smad proteins [55], [56] we investigated if *fuss* is able to inhibit the TGF-β/BMP cascade, which is represented in Drosophila by BMP/Activin-β signaling. We made use of the wing blade and the adult wing, an amenable and widly used tissue to analyse function and crosstalk of members within this signaling pathway. The ectopic expression of *fuss* in the wing affects the overall vein structure (Fig. 3B/C). While *fussC* produces defects in L2, L5 and p-cv and results in a loss of the campaniform sensilla on L3, the *fussB* isoform overexpression leads to a complete wing disruption or poorly unfolded extremities in the few female escapers which hatch. Together these phenotypes are highly reminiscent of loss of function phenotypes within the *dpp* pathway and provoked us to investigate if and how *fuss* is able to interact negatively within this cascade. While the contribution of BMP signaling to *Drosophila* development is enormous, including cell-fate specification, imaginal disk patterning or growth organization, the Activin-ß branch has only been elucidated recently. It could be shown, that its components regulate neuronal wiring and proliferation, mushroom body remodeling and the morphogenesis of neurons in the adult [47], [57]. We were wondering if we can decipher the pathway affected by *fuss* overexpression and examined the expression pattern of prominent read-outs, namely *omb* and *sal* for BMP signaling and *EcR1B* for the Activin-ß cascade. Our results show a clear reduction of *omb* and *sal* on a histological and also molecular level, which leads us to the conclusion, that *fuss* is indeed an inhibitor of the BMP pathway. Moreover, the coexpression of *dpp*-cascade activators like the typeI receptor *saxophone* or the Smads *mad* and *medea* with *fuss* results in a clear rescue of vein patterning and wing size. In the case of *medea* we observed an almost complete rescue of the *A9-Gal4;UAS-med* wing phenotype and postulated a direct interaction of *fuss* with *medea* via its SMAD4 binding domain (Fig. 2A), like it has been described for *dSno* [29] or *c-ski* [55]. In contrast to the BMP pathway, no effects could be observed on the expression of one of the main target genes of the Activin-ß pathway, *EcR1b.* This result was further supported by a failure to detect genetic interactions of *fuss* with members of the Activin-ß branch, which further supports a specific inhibitory function of *fuss* on the BMP pathway.

To further strengthen our hypothesis of a specific interaction of Fuss with Medea, the direct interaction of these two proteins was identified by a yeast two hybrid experiment and confirmed by CoIP in *Drosophila* cell culture. Interestingly we could not detect an interaction between Fuss and Mad *in vitro*, although the genetic interaction of Fuss with Mad revealed a partial rescue of the wing phenotype. The fact that pMad concentration in wing disks is not reduced in the presence of Fuss clearly indicates, that Fuss function is downstream of R-Smad activation. One possibility could be that Fuss is able to titrate out pMad/Med or possibly forms a trinary complex with pMad/Med affecting BMP target gene expression like *omb*.

Is there a functional difference between *dSno* and *fuss*, both belonging to the ski family? Although *dSno* exerts its effects also through *med,* it is supposed to act as a BMP-to-Activin-ß pathway switch, at least in brain development. By forming a complex with dSMAD2 it directs differentiation of neuroblasts towards proliferation [29], [31], a role we can not ascertain for *fuss*. However, we can detect a clear difference in vein patterning defects comparing dSno and fuss overexpression using an identical driver line (*A9-Gal4*) further supporting an individual and different inhibitory effect of the closely related ski/sno/fuss proteins.

The interaction of *fuss* with *med* and the subsequent inhibitory effects on BMP signaling, led us to the assumption, that the subcellular localization of Fuss might undergo changes during *dpp* activation transmitted by *med* overexpression. In general, the subcellular localization of homologous proteins such as Ski and SnoN is variable and depends on several conditions, such as morphological differentiation of cells or activity of proteins in normal versus tumor tissues; for example SnoN localization in nontumorigenic cells is preferentially cytoplasmic, while in tumor cells it is constitutively nuclear [58]. Our GFP tagged Fuss protein is predominantly localized in the cytoplasm, when it is overexpressed by itself. Here it might sequester Med and prevent its nuclear translocation in response to *dpp* signaling or another yet unidentified factor. However, overexpression of *med* together with *fuss* leads to a clear relocalization from the cytoplasm into the nucleus. Fuss thereby antagonizes the BMP cascade, which is overstimulated by excessive med signaling leading to an almost complete rescue of the med overexpression wing phenotype. Interestingly, we observed, that pMad still translocates into the nucleus upon *fuss* overexpression. Considering the genetic interaction results, it is very likely that pMad/Med/Fuss enter the nucleus already as a trimer, which then might lead to a change in DNA binding or regulatory capabilities of the Smad complex.

Our results also need to be discussed in respect to recent data on the mammalian Fussel genes, in particular the isolation and characterization of a transposon induced mouse null allele of Fussel-18 (Skorl-2) [41]. Interestingly one prominent signaling phenotype in these mice is a strong repression of sonic hedgehog (Shh) signaling. In particular the authors could show that Fussel-18 is able to bind R-Smads and Co-Smads leading to a specific reduction of BMP- but not TGF-β-signaling. This nicely fits our genetic interaction results, which show that both Mad and Med interact with Fuss, although we could only show physical protein interaction for Med and not for Mad. The Shh repression effect in the mouse mutant can be explained through a repressing function of BMP signaling on Shh, which in a wildtype background, is repressed by Fussel-18 itself, reestablishing Shh expression [41].

The exact mechanism through which *fuss* exerts its endogenous function remains to be elucidated. As mentioned before, the protein exhibits a DHD motif, known to be responsible for DNA binding. Although a direct interaction with DNA could neither be shown for the human Ski-Sno proteins, nor for *Drosophila* SNO [29], [37], [59] we can not rule out the possibility that *fuss* translocates to the nucleus (with or without *med*) and binds itself to DNA. Yet we rather propose an association of Fuss with other protein partners, such as the corepressors *Smrter* or *dSin3A* or that it forms a complex with the histone deacetylase *Rpd3*, such an association of Fussel-18 with HDAC1 has been described in the mouse [41]. It is also possible that *fuss* displaces coactivators, such as the *Drosophila* homolog of the p300/CBP complex called *nejire* [60]–[62] or stabilizes inactive SMAD complexes [55].

Further investigations will elucidate the endogenous role of *fuss* during *Drosophila* development and the processes by which it antagonizes BMP signaling.

## Materials and Methods

### Fly Strains and Drosophila Genetics

Flies were kept under standard conditions. All Gal4-lines, except of *nub-Gal4* (J.F. deCelis, Madrid), were obtained from the Bloomington Stock Center, the *UAS-fussC-IR*-lines (transformant IDs: 11093R-1 and 11093R-3) were obtained from the National Institute of Genetics, Japan and the Vienna Drosophila RNAi Center (transformant ID 15478). Other UAS-constructs used were: *UAS-SaxA* [52], *UAS-Mad* [63], *UAS-Med* [64], and UAS-babo* [65]. To create the *fuss* overexpression constructs, the full-length *fussC* cDNA-clone was obtained from the *Berkeley Drosophila Genome Project* (IP13014), excised from pOT2 and directly cloned into pUAST using the BglII and XbaI restriction sites. The 5′- region of *fussB* cDNA was isolated from CantonS flies by RT-PCR using 5′-ATGGATTTAAATGAAAATTTTAAAA-3′/5′-AACGCAAAGTCGTCAGGTAG-3′ primers and T/A- cloned in pGEMT-Easy (Promega, WI). Subsequently, a BglII restriction site and an invertebrate Kozak consensus sequence (CAAA) were added to the 5′ end of the fragment by PCR and exchanged with the 5′ part of the *fussC*-cDNA in the pUAST construct. For the fussel-GFP-fusion, the stop-codon in *fussC* was mutated by PCR (5′-TCTAGAGACTAGACTATTTTTATTTCCAGAAG-3′; Stop → ArgSer) and after ligation with the 5′ end of eGFP using the introduced XbaI restriction site cloned into pUAST. The transformation vectors were injected into syncytial yw; Δ*2–3, Ki* embryos.

### Expression Analysis

cDNA was produced by extraction of RNA from one hundred embryos, ten L3-larvae, ten praepupae, ten pupae or ten adult CantonS flies using *peqGOLD Trifast* (Peqlab, Germany) and reverse transcription with *QuantiTect Reverse Transcription Kit* (Quiagen, Germany). Primers used for the confirmation of the predicted 5′- ends of the transcripts by PCR were 5′-ATGCCAGTGAGTTCCCGACGAT-3′/5′-AACGCAAAGTCGTCAGGTAG-3′ for *fussC* and 5′-ATGGATTTAAATGAAAATTTTAAAA-3′/5′-AACGCAAAGTCGTCAGGTAG-3′ for *fussB* respectively. For the confirmation of the 3′ splice sites, primers were 5′-ACGAGTCCCATTCCTCAA-3′/5′-CTACTACTTCGTCGTCATC-3′ spanning the intron between exon 3 and 4 in *fussB/fussC* and 5′-GACGACGAAGTAGTAGACA-3′/5′-CTTATTGGACTCCGCCAC-3′ spanning the intron between exon 4 and 5 in *fussB/fussC*.

### Real Time PCR

cDNA samples from three different individual crosses were tested in the *Lightcycler*- System (Roche, Switzerland) using the *QuantiTect SYBR Green* RT- PCR- Kit (Qiagen, Germany). For relative quantification, we applied the delta- delta CT algorithm. Primer pairs used for the experiments were: *rp49*: 5′-GCGGGTGCGCTTGTTCGATCC-3′ and 5′-CCAAGGACTTCATCCGCCACC-3′, *fuss:* 5′-AGTTGGAGTAACGGCGGTAG -3′ and 5′-TTGGGTAAGGCTGCTGATA-3′, *omb* 5′-ACTGGGCACGGAAATGGT-3′ and 5′-GGGCGAATCTGGATGGATAT-3′, *salm:* 5′-CCACCGCCAAGATGCTAT-3′and 5′-CGATGAAGTTCTCCCACGA-3′, *ecr1b*: 5′-GCACCTGGTTCCTTGTCC-3′ and 5′-TCTGGGCGTTCGCATACA-3′.

### Yeast Two-Hybrid

The Yeast two hybrid tests were performed as described in the instruction manual from PROQUEST (Life Technologies, CA) using the full-length *fussC* cDNA cloned into pDBLeu. For the direct two-hybrid tests with *medea* and *mad,* the full length cDNAs LD22279 and RE72705 were cloned into pPC86.

### Co-immunoprecipitation

*fussC*, *medea* and *mad* were amplified and 5′BglII or 5′BamHI and 3′ XbaI restriction sites were appended by PCR. After T/A-cloning in pGEM-T Easy, the coding sequences were transferred in the expression vectors pFSR-HA and pFSR-FLAG (F. Sprenger, Regensburg, Germany) using the BamHI and XbaI sites. *S2* cells were grown in Schneider’s *Drosophila* Medium supplemented with 10% heat-inactivated fetal bovine serum (Pan Biotech, Germany) and transfected at 90% confluency in 6-well plates using FuGeneHD transfection reagent (Roche, Switzerland). Cells were harvested after 48 hours of induction with 1 mM CuSO_4_, washed with PBS and lysed in ES2 cell lysis buffer (20 mM HEPES, pH 7.5, 50 mM KCl, 2.5 mM EDTA, 5 mM DTT, 0.05% Triton X-100, 5% Glycerol) supplemented with Complete Mini protease inhibitor cocktail (Roche, Switzerland). After removal of the cell debris and pre-clearing with Protein-G Sepharose (Sigma-Aldrich, MO) the lysates were incubated for four hours with anti-Flag M2 antibody at 4°C (Sigma-Aldrich, MO). To precipitate the complexes, Protein-G Sepharose was added and the mixture was incubated for another two hours at 4°C. After four washes, the precipitates were resuspended in ES2 supplemented with the protease inhibitor cocktail. The cell lysates and precipitates were analysed by standard SDS-PAGE followed by western blotting. The blots were blocked and incubated overnight with anti-FLAG M2 or anti-HA 12CA5 (Abcam, UK) primary antibodies and Alexa Fluor 680 goat anti-mouse A-21057 (Life Technologies, CA) secondary antibody. Signals were detected using an Odyssey infrared imaging system (Li-Cor, NE).

### Analysis of Wing-venation and Size

Wings were mounted on microscope slides in DePeX and fixed under a glass cover slide. Digital pictures were taken on an Axiophot Microscope (Zeiss, Germany) at a resolution of 1360×1024 Pixels. Overall wing size was measured in ImageJ 1.44e using the *‘Huang’* thresholding algorithm to create binary pictures. The function ‘*analyse particles’* with activated *‘include holes’-* option was used to measure the size of the wing in pixels.

### RNA *in situ* Hybridisation, X-Gal-staining and Immuno-histochemistry

RNA *in situ* hybridization on embryos and third instar larval brains were carried out according to Tautz and Pfeifle (1989) [66]. The *fuss* sense and anti-sense digoxigenin-labeled RNA probes were prepared with T7 and SP6 RNA polymerases using the *fu*ss*C* cDNA as template with the following primers: 5′-ATCAGCAGAAGGAAAATTGAAAAGGTAAGC-3′ (for) and 5′-CATCGTAATCATTTCCACTCAGAGAC-3′ (rev). For signal detection, alkaline phosphatase-conjugated anti-digoxigenin antibody and NBT/BCIP stock solutions (Roche, Switzerland) were used. For the X-Gal stainings, larvae were dissected in ice cold PBS and fixed with 1% Glutaraldehyde. The tissue was incubated for 2 hours at 37°C with 0,4% X-Gal in staining solution (10 mM Phosphate Buffer pH 7,2, 150 mM NaCl, 1 mM MgCl, 3 mM tetrapotassium hexacyanoferrate, 3 mM tripotassium hexacyanoferrate, 0,3% Triton X-100), washed in PBS and incubated at 4°C in 70% glycerine/PBS over night before mounting in glycergel. For immunohistochemistry, L3 wing disks were dissected in PBS and fixed for 1 h in 4% paraformaldehyde in PBT (PBS containing 0,1% Triton X-100). Tissues were washed five times in PBT and blocked 2 h with 10% serum in PBT. Primary antibody incubation was carried out over night at 4°C in blocking solution, followed by five washes in PBT and 4 h incubation with the secondary antibody at room temperature in blocking solution. After five washes in PBT, disks were mounted in *VectaShield* (Vector Labs, CA) or glycergel. Primary antibodies anti-dSRF 39093 (Active Motif, CA), anti-phospho-SMAD1/5 #9516 (Cell Signaling, MA) anti-GFP A-6455 (Invitrogen, CA) and anti-histone ga199 (A. Hofbauer, Regensburg, Germany) were used at 1∶250 dilutions. Secondary goat anti-mouse-AF4568 A-11031, goat anti-rabbit-AF488 A-11034, goat-anti-mouse-AF647 A-21236 (Invitrogen, CA) and biotinylated goat anti-mouse BA-9200 (Vector Labs, CA) were used at 1∶500 dilutions. The tissue was analysed on a LSM 510 META confocal microscope or an Axiophot Microscope (Zeiss, Germany).

### Bioinformatics

For the identification of proteins similar to FussB and FussC the UniProtKB Database (http://www.uniprot.org) was analyzed using BLAST. Protein sequences with significant sequence similarity to FussB or FussC were aligned in Clustal-X 2.0 [67]. The unrooted phylogenetic tree and the estimate significance of branch points were calculated in MEGA4 [68] using the neighbour-joining method with bootstrap resampling. Coils 2.2 [69] was used for the identification of coiled coil motifs.
